# Child Welfare in Manchester

**Published:** 1920-06-26

**Authors:** 


### Child Welfare in Manchester.

Last week we gave some account of the plans and experiences in welfare work at Lincoln, as an example of what is being done in the smaller boroughs. This week there are available some facts of the organisation as carried out in a big city like Manchester, on whose behalf it is claimed by the *Manchester Guardian* that, in taking advantage of the powers conferred by the Child Welfare Act, it has probably been more successful than any other town in the provinces. According to the figures given in this paper, the death-rate in 1919 among infants under one year was one-fourth of what it was in 1913. It has shown a very steady decrease for several years past owing to the efforts made by the Public Health Department. There are now eleven Welfare Centres under the Corporation of Manchester, and each is under the supervision of a fully-trained nurse who is also a certified midwife. Some have also the services of a trained masseuse, and there are three medical officers who devote full-time service to the centres. At two of the centres there are V.D. clinics, where mothers are treated before the birth of the child and afterwards. Milk is supplied to expectant and nursing mothers, in necessitous cases free and in other cases at a very cheap rate. Children whose parents are in very poor circumstances are given either fresh or dried milk and any necessary supplementary food, the amount being decided by the medical officer.

Babies are brought to be weighed daily, and at one centre it is quite usual to see between eighty and ninety babies in one afternoon. In this part of the work the superintendent is assisted by voluntary workers. Young mothers may also receive advice on the clothing of their infants. Garments are cut out and made up. The health of each baby is carefully observed, and in the case of any signs of constitutional ill-health or disease the mother is advised to take her infant to her own doctor. Thus the centres do not interfere with the interests of the medical practitioner; in fact they are usually of assistance to him.

There are forty-six health visitors employed by the Corporation, and with the exception of four these are all fully-trained nurses. The other four are widely experienced. These visitors are welcomed by the people in the most friendly way. The baby comes under the observation of the health visitor from the twelfth day after its birth. Up till that time both the mother and child are cared for by the midwife. By means of visiting the homes those in charge of the centres become acquainted with the conditions and circumstances of individual families, which is of great assistance in their observation of the children.

This part of the work of the Public Health Department in Manchester is constantly increasing. More districts are gradually being covered by the activities of the Centres, but there are still many evils that will take a great deal of time to do away with. Experimental work is constantly being done, and when this is found to be successful it becomes a permanent part of the scheme. It is within the power of the citizens of Manchester to help the Corporation very much in the great work of reclaiming the children, and the baby week campaign in which Manchester is taking part should do much to make known the valuable work and the directions in which it needs extension and help.

